# Long-Lived Charge-Separated States in Self-Assembled TiO_2_ Photoanodes Incorporating a Spin-Transition Cobalt Complex

**DOI:** 10.1021/acs.jpcc.6c00432

**Published:** 2026-03-26

**Authors:** Tzu-Ching Cheng, Vasily Vorobyev, Savannah Pearson, Niroshani S. Abeynayake, Carla Slebodnick, Zhichun Shi, Amanda J. Morris

**Affiliations:** † Department of Materials Science & Engineering, Virginia Tech, Blacksburg, Virginia 24061, United States; ‡ Department of Chemistry, Virginia Tech, Blacksburg, Virginia 24061, United States

## Abstract

Improving the efficiency of photoelectrocatalytic cells relies on precise control of interfacial electron transfer rates to favor the generation of long-lived charge-separated states. Achieving efficient forward electron transfer while suppressing charge recombination remains a central challenge. In this study, we investigate a cobalt-based transition metal complex that undergoes charge transfer-induced spin crossover (CTISC) as a strategy to modulate interfacial charge dynamics in dye-sensitized photoelectrochemical architectures. Ultrafast and nanosecond transient spectroscopy were used to quantify electron injection and dye regeneration. Open-circuit voltage decay measurements were employed to assess the lifetimes of injected electrons under operating conditions. Density functional theory (DFT) calculations were used to estimate the inner-sphere and outer-sphere reorganization energies associated with the redox processes. The results demonstrate that the large inner-sphere reorganization energy associated with spin-state change significantly prolongs charge-separated lifetimes. These findings highlight the potential of spin-state-mediated reorganization as a design principle for suppressing charge recombination and improving the performance of dye-sensitized photoelectrochemical systems.

## Graphical abstract

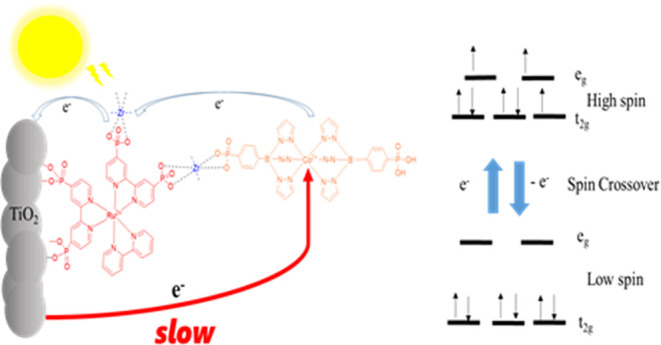

## Introduction

Harnessing solar energy to produce chemical fuels presents a promising strategy for generating sustainable, high-energy-density energy carriers. In nature, the process is exemplified by photosynthesis, in which light-driven water oxidation is coupled with carbon dioxide reduction to form energy-rich sugars. Artificial photosynthetic approaches aim to replicate these fundamental transformations, primarily through the water oxidation reaction (WOR) and the hydrogen evolution reaction (HER).^1^ One such platform is the dye-sensitized photoelectrochemical cell (DSPEC), in which light absorption by a molecular chromophore followed by electron injection into a semiconducting metal oxide generates a charge-separated state capable of driving redox chemistry at proximal catalytic sites.

Despite significant progress, the performance of DSPECs remains limited, with efficiencies typically in the 0.1%–1% range, and the highest reported value of 4.15% solar-to-fuel conversion efficiency.^2–5^ The modest efficiencies largely arise from rapid charge recombination between injected electrons and oxidized chromophores or catalysts, which competes directly with productive chemical transformation. Consequently, considerable effort has been devoted to extending the lifetimes of charge-separated states. Strategies include the thermodynamic adjustment of chromophores or catalysts^6,7^ and spatial separation between metal oxides and catalysts.^8,9^

An alternative strategy to increase charge-separated state lifetimes involves the use of charge-transfer-induced spin crossover (CTISC) compounds, in which electron transfer is coupled to a change in spin state that introduces a substantial inner-sphere reorganization energy. The reorganization process can kinetically impede back electron transfer, thereby extending charge-separated lifetimes beyond what is achievable through electronic or spatial effects alone. The first demonstration of CTISC-mediated long-lived charge-separated states was reported in a dye-sensitized solar cell (DSSC) employing a cobalt­(II/III) tris­(bipyridyl)-based redox mediator.^10^ In the cell, the oxidation of high-spin, quartet Co­(II) to low-spin, singlet Co­(III) was accompanied by a substantial geometric rearrangement (shortening of the Co–N coordination bonds), resulting in a large inner-sphere reorganization energy that kinetically inhibited charge recombination. However, Co­(II) tris­(bipyridyl) complexes exist as a mixture of high-spin species (60%) and low-spin species (40%) at room temperature.^11,12^ The mixed-spin nature of Co­(II) complicates the direct assessment of the role of CTISC in suppressing recombination.

To address this limitation, we explored a series of manganese poly­(pyrazolyl)­borate complexes, [Mn­(Tp)_2_].^13,14^ The spin state of Mn­(II) is a high-spin, sextet state, and Mn­(III) exhibits a low-spin, triplet state. Unlike Co­(II) tris­(bipyridyl) complexes, the [Mn­(Tp)_2_] compounds display a clean and well-defined CTISC behavior. Incorporation of the complexes into quantum dot and dye sensitized photoelectrodes demonstrated that CTISC can significantly extend charge-separated lifetimes, validating the design principle for photoelectrochemical applications.

The incorporation of covalently linked chromophores and electron-donating moieties is a widely employed strategy to enhance chromophore regeneration in DSPECs.^15,16^ In the approach, intramolecular electron transfer from a tethered donor to an oxidized chromophore can outcompete deleterious back-electron transfer to the semiconductor. Metal-ion-mediated self-assembly offers a particularly attractive platform for constructing these architectures, providing modularity and synthetic simplicity compared to covalently preassembled systems.^17–19^

Herein, we report the integration of a CTISC-active cobalt complex in a self-assembled photoanode construct.^20^ A TiO_2_-bound, phosphonate-functionalized chromophore was coupled to [Co^II^(4-PO_3_H_2_PhB­(pz)_3_)_2_] (Co­(II)) through a Zr^4+^-bridge, Figure 1. The resulting construct enables direct interrogation of how CTISC influences charge recombination in a surface-bound architecture. Ultrafast and nanosecond transient absorption spectroscopy, combined with open-circuit voltage decay measurements, were used to investigate charge separation and recombination dynamics. Complementary density functional theory (DFT) calculations were used to quantify the associated reorganization energies. Overall, the results demonstrate that incorporation of a CTISC-active donor leads to a notable increase in charge-separated lifetime (30%) compared to a neat sensitized photoanode. The work establishes a new framework for integrating spin-state control into molecular photoelectrochemical systems and highlights the potential for CTISC-based complexes to advance solar fuel generation.

**Figure 1:**
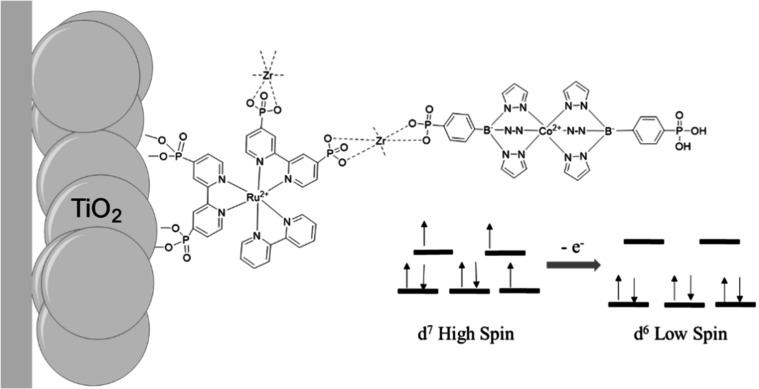
Scheme of the as-synthesized self-assembled photoanode, TiO_2_/Ru­(II)­P2–Zr^4+^-Co­(II).

## Experimental Methods

### Materials

Diethyl phosphite (≥98%), tetrakis­(triphenylphosphine)­palladium(0) (Pd­(PPh_3_)_4_, ≥99%), zinc granules (≥99.8%, 20–30mesh), pyrazole (98%), sodium hydride (60% dispersion in mineral oil), 4-bromophenylboronic acid (≥95%), thallium acetate (≥99%), cobalt­(II) chloride hexahydrate (98%), and bromotrimethylsilane (97%) were purchased from Sigma-Aldrich. 4,4′-dibromo-2,2′-bipyridine (98%) was purchased from Oakwood Chemical. Ruthenium­(III) chloride trihydrate (95%) was purchased from Ambeed. Diethylene glycol dimethyl ether (diglyme, >99%) was purchased from TCI America. All solvents and chemicals were used as received without further purification unless indicated otherwise.

### Synthesis of [Ru­(bpy)­(4,4′-(PO_3_H_2_)_2_bpy)_2_]­Cl_2_ (Ru­(II)­P2) Synthesis

The synthesis of photosensitizer (Ru­(II)­P2) was modified from Norris et al.^21^ The ligand 4,4′-bis­(diethylphosphonate)-2,2′-bipyridine was synthesized by reacting diethyl phosphite (2.3 equiv), 4,4′-dibromo-2,2′-bipyridine, (1 equiv), and Pd­(PPh_3_)_4_ (0.1 equiv) under Schlenk conditions. Anhydrous toluene and triethylamine were then added, and the reaction mixture heated to 110 °C for 4 h. The reaction mixture was filtered hot, and the solvent was removed under vacuum. The crude product was recrystallized from hexanes to give light yellow needles. The recrystallization was repeated at least three times.

To prepare the *cis*-[Ru­(4,4′-(PO_3_Et_2_)_2_bpy)_2_Cl_2_], RuCl_3_·3H_2_O (1 equiv), 4,4′-bis­(diethylphosphonate)-2,2′-bipyridine (2 equiv), and zinc granules (3.5 equiv) were refluxed in ethanol (*C*_Ru_ = 7 mM) for 12 h. The reaction mixture was filtered hot, and the solvent was removed from the filtrate under vacuum. The resulting dark-purple solid was collected, washed with diethyl ether, and dried. Further purification was performed through silica gel flush column.

To form [Ru­(bpy)­(4,4′-(PO_3_Et_2_)_2_bpy)_2_]­Cl_2_, 2,2′–bipyridine (3 equiv) was reacted with *cis*-[Ru­(4,4′-(PO_3_Et_2_)_2_bpy)_2_Cl_2_] (1 equiv) in absolute ethanol in a Teflon vessel under microwave heating at 150 °C for 40 min. After cooling to room temperature, ethanol was removed under vacuum. The diethyl phosphate esters were hydrolyzed to phosphonic acid using concentrated HCl. The product was purified by silica gel chromatography and extracted with dichloromethane/water three times to remove excess 2,2′–bipyridine. Yield: 11%. ^1^H NMR (400 MHz, D_2_O) 8.84 (d, 4H), 8.51 (d, 2H), 8.14 (t, 2H), 7.99 (m, 4H), 7.83 (d, 2H), 7.65 (dd, 4H), 7.46 (t, 2H).

### Synthesis of [Co­(4-PO_3_H_2_PhB­(pz)_3_)_2_]

The ligand precursor, thallium­(4-bromophenyl)­tris­(pyrazolyl)­borate, was synthesized according to the Faller and White^22^ Pyrazole (1.1 equiv) was deprotonated with sodium hydride (1 equiv) in diglyme (0.16 M). Additional pyrazole was added as needed to ensure complete consumption of sodium hydride. 4-Bromophenylboronic acid (1 equiv) and pyrazole (2 equiv) were then combined and the reaction was heated to 160 °C under nitrogen for 2 h. After cooling to 55 °C, water was added to form a milky-colored solution. The solution was then treated with activated carbon and stirred overnight at room temperature. The filtrate was collected via vacuum filtration. The filtrate was treated with thallium acetate (1 equiv) to precipitate thallium (4-bromophenyl)­tris­(l-pyrazolyl)­borate (Tlpz), which was collected by centrifugation and dried under vacuum. Yield: 22%. ^1^H NMR (400 MHz, CDCl_3_) 7.65 (d, 3H), 7.49 (dd, 2H), 7.44 (d, 3H), 7.44 (d, 3H), 7.03 (d, 2H), 6.28 (t, 3H).

The cobalt complex, [Co­(4-BrPhB­(pz)_3_)_2_], was prepared by stirring Tlpz (2 equiv) and CoCl_2_·6H_2_O (1 equiv) in a pH 5.2 buffer solution/acetone mixture (3:1) at room temperature for 1 h. The product was extracted with DCM/water three times to remove excess metal salt. Single crystals were obtained by slow evaporation of the solution in DCM. Yield: 31%.

The substitution of –Br with –PO­(OEt)_2_ was completed with Hirao coupling with a Pd­(PPh_3_)_4_ catalyst.^23^ Diethyl phosphate (2 equiv), [Co­(4-BrPhB­(pz)_3_)_2_] (1 equiv), Pd­(PPh_3_)_4_ (0.6 equiv) were refluxed in anhydrous THF (24 mM) for 2 days. Once cooled to room temperature, the solvent removed via vacuum filtration. The crude product was purified with silica gel chromatography to remove the Pd­(PPh_3_)_4_ catalyst. Yield: 36%.

Bromotrimethylsilane (6 equiv) was used in anhydrous MeCN (*C*_Co_ = 5 mM) for the hydrolysis^24^ of [Co­(4-PO­(OEt)_2_PhB­(pz)_3_)_2_] to [Co­(4-PO_3_H_2_PhB­(pz)_3_)_2_]. The reaction was stirred for 2 days under N_2_ flow. The solvent was removed under vacuum. Reversed-phase silica gel chromatography was performed to remove other partially hydrolyzed products. The obtained elution was recrystallized in methanol via slow evaporation in air. The obtained single crystal was found to be [Co^III^(4-PO_3_H_1.5_PhB­(pz)_3_)_2_]·2H_2_O.

A solution of Na_2_S_2_O_4_ (1 equiv) in water/methanol mixture (10:1) was used to reduce [Co^III^(4-PO_3_H_1.5_PhB­(pz)_3_)_2_] to [Co^II^(4-PO_3_H_1.5_PhB­(pz)_3_)_2_]. After stirring overnight at room temperature, the solution was dried under vacuum. The desired cobalt complex was recrystallized from methanol with filtration to eliminate sodium salts. The oxidation state Co^II^ was further confirmed with XPS. Yield: 18%. (*m*/*z* calculated: 797.1487, found: 797.1401 [M]^+^).

### Electrochemical Measurements

Electrochemical experiments were conducted in a three-electrode configuration using a glassy carbon working electrode, platinum mesh counter electrode, and Ag/Ag^+^ reference electrode (0.01 M AgNO_3_). Measurements were performed in methanol containing 0.1 M TBAPF_6_. Electrodes were polished prior to each experiment, and all potentials were referenced to the Fc/Fc^+^ couple.

### X-ray Photoelectron Spectroscopy

XPS measurements were performed on a PHI VersaProbe III instrument using monochromatic Al Kα radiation (1486.6 eV). The analysis area was 1000 × 1000 μm^2^. Spectra were calibrated using the C 1s peak.

### Crystal Structure Determination

Diffraction data was collected either at ORNL using a Rigaku XtaLAB Synergy-DW diffractometer equipped with a HyPix-ARC 150 detector and operating with Cu Kα radiation ([Co­(4-PO_3_H_2_PhB­(pz)_3_)_2_]) or on an in-house Synergy-S diffractometer equipped with a HyPix-6000HE detector ([Co^II^(4-BrPhB­(pz)_3_)_2_]) and operating with Mo Kα radiation. The data collection routine, unit cell refinement, and data processing were carried out with the program CrysAlisPro.^25^ The structures were solved with SHELXT^26^ and refined with SHELXL^27^ via Olex2.^28^

[Co­(4-PO_3_H_2_PhB­(pz)_3_)_2_]­(0.02 × 0.10 × 0.12 mm^3^) The triclinic space group *P*1̅ was assigned. The final refinement model involved anisotropic displacement parameters for non-hydrogen atoms. A riding model was used for the C–H hydrogen atoms. The O–H hydrogen atoms were located from the difference electron density map and the positions and isotropic displacement parameters were refined independently. Atom H2 originally appeared on an inversion center. A PART −1 instruction allowed this H atom to move off the symmetry constrained position and model as 50/50 disordered across the symmetry equivalent O2 atoms to give 50% RPO_3_H and 50% RPO_3_H_2_.

[Co^II^(4-BrPhB­(pz)_3_)_2_] (0.14 × 0.18 × 0.34 mm^3^): The Laue symmetry was consistent with the triclinic space groups *P*1 and *P*1̅. Although the E-statistics favored *P*1, the structure was originally solved in *P*1̅. This structure model exhibited many problems including unreasonable anisotropic displacement parameters, solvent that could not be located (a solvent mask was used), unusually poor refinement statistics for the quality of the data, and large peaks and holes in the difference electron density map. In space group *P*1, the problems observed with *P*1̅ were resolved. The unit cell comprises two metal complexes and three CH_2_Cl_2_ solvates. The metal complexes have pseudo inversion symmetry with 97% of the unit cell contents related by inversion symmetry. Three CH_2_Cl_2_ molecules break the inversion symmetry. No disorder model was needed for the CH_2_Cl_2_. The final refinement model involved anisotropic displacement parameters for non-hydrogen atoms and a riding model for all hydrogen atoms. The material crystallizes as a racemic twin (BASF = 0.499(4)).

### Self-Assembled Layer Preparation

Self-assembled layers were prepared in a procedure similar to previous literature.^14,17^ The glass slides or fluorine-doped tin oxide (FTO, 10 Ω/sq Hartford glass) were cut to fit the diagonal of an optical cell (for spectroscopic measurements). The TiO_2_ layer was deposited via a doctor blade method using a commercial transparent TiO_2_ paste (T/SP, 100% anatase, 15 nm–20 nm from Solaronix). The deposited TiO_2_ films were dried in an oven at 130 °C overnight before being calcined at 500 °C for 1 h. To prepare the Ru­(II)­P2 layer, the TiO_2_ slides were immersed in the saturated Ru­(II)­P2 in methanol. When the λ_max_ of UV–vis spectra of the thin film reached extinction coefficient 0.8, the Ru­(II)­P2 layer was considered complete. The surface coverage on TiO_2_ for Ru­(II)­P2 was Γ = 6.4 × 10^–7^ mol·cm^–2^. The thin film was rinsed with methanol three times and dried under a flow of nitrogen. ZrOCl_2_ was used as a bridge to connect photosensitizer and electron donors.^29^ ZrOCl_2_ (0.1 M) was dissolved in methanol, and a TiO_2_/Ru­(II)­P2 thin film was immersed overnight. The TiO_2_/Ru­(II)­P2–Zr^4+^ thin film was immersed overnight in a methanolic solution of [Co­(4-PO_3_H_2_PhB­(pz)_3_)_2_] to incorporate the complex into the film, resulting in TiO_2_/Ru­(II)­P2–Zr^4+^-Co­(II).

### Photoanode Characterization

To characterize the thin film composition (photosensitizer, metal ion bridge, and electron donors), inductively coupled plasma mass spectrometry (ICP-MS) was used. For ICP-MS sample preparation, the assemblies were desorbed from the surface using 3 mL of 0.1 M NaOH in HPLC-grade water for 2 h.^30^ The resulting liquid was sent to Galbraith Laboratories, Inc. (TN, USA) using GLI procedure ME-30. The detection limits for Ru, Zr, and Co are 0.05, 0.1, and 0.1 μg/L, respectively.

### Time-Resolved Transient Absorption Spectroscopy

Nanosecond transient absorption spectra were collected using an enVISion UV/Visible/Near-IR Transient Absorption Spectrometer (Magnitude Instruments, PA, USA). The excitation source was a 532 nm second harmonic generated from a Nd:YAG laser. The repetition rate during measurement was set to 400 Hz with a mechanical chopper. The energy of the pulse is 0.04 mJ/pulse. Samples were prepared by placing a thin film into a 1 cm optical cell diagonally fitted with a 24/40 ground glass joint. An electrolyte solution consisting of 0.1 M LiClO_4_ in anhydrous acetonitrile was then added to the optical cell. The cell was sealed using a 24/40 rubber septum, and the joint was wrapped with electrical tape. Removal of oxygen was achieved using a double purging apparatus to prevent the loss of acetonitrile. The prepurging flask was purged for 30 min with argon before connecting the optical cell, which was purged for an additional 30 min. The stability of each sample was assessed by measuring the UV Vis spectra of the thin film before and after each experiment.^14^

### Ultrafast Transient Absorption Spectroscopy

Femtosecond transient absorption measurements were conducted using a pump–probe technique based on a 1 kHz Ti:sapphire chirped pulse amplifier (Astrella-F-1K one-box femtosecond amplifier). The 400- and 500 nm pump pulses were produced from an Optical Parametric Amplifier (OPA, Apollo, Ultrafast Systems). The energy of the pump beam was measured after the chopper operating at a 500 Hz repetition rate with a Thorlab power meter PM100D equipped with a thermal head S401C. A variable ND filter was used to tune the laser power intensity, and the pump intensity was measured to be 1.82 μJ/pulse for 400 nm excitation light and 1.20 μJ/pulse for 500 nm excitation light. The delay line, pump beam focusing lens, adjustment mirrors, and white-light generation stage are integrated into the Helios Fire instrument from Ultrafast Systems. The probe beam, after passing through the sample, was collected using a pair of parabolic mirrors and focused into a fiber, which transmitted the light to the spectrometer equipped with a CCD camera system from Princeton Instruments.

### Open-Circuit Voltage Decay Measurements (OCVD)

The OCVD measurements were performed in a manner similar to that described in previous literature.^14^ A Newport LCS-100 solar simulator (AM 1.5G spectrum) with an incident light intensity of 0.2 Sun was used. The electrolyte was 0.1 M LiClO_4_ in MeCN. The open-circuit potential was monitored using a Pine Instruments WaveNow potentiostat at 5 ms intervals.

### DFT Calculations

Calculations of the optimized geometries and single point (SP) energies for the structure of [Co^II/III^(4-PO­(OMe)_2_PhB­(pz)_3_)_2_]^0/+^ were performed using the Gaussian 16-C.02 program package.^14^ Geometry optimizations were carried out with the BP86 functional using the 6–31G­(d) basis set for all atoms except Co and the standard double-ζ-type LANL2DZ basis set with the effective core potential of Hay-Wadt for Co. The computations of the SP energies were performed at the B3LYP/6–31G­(d) level of theory with the same exception of Co atom. All calculations were carried out by using the conductor-like polarizable continuum model (CPCM) with the default parameter for acetonitrile. Details of solvation shell of an optimized structure with correctly assigned charge and spin states were saved and used for SP calculations of nonequilibrated states with the preserved geometry but different spin and charge states. These energy terms were used to calculate the total reorganization energy. The inner sphere reorganization energy was calculated from the energy terms obtained in SP calculations with the optimal solvation shells, which were constructed de novo for both optimal and nonoptimal spin/charge states.

## Results and Discussion

The target CTISC complex [Co­(4-PO_3_H_2_PhB­(pz)_3_)_2_] was prepared via Hirao coupling to form the corresponding ester, followed by hydrolysis with bromotrimethylsilane.^23^ Recrystallization afforded single crystals suitable for X-ray diffraction (Figure S4). The phosphonate groups were modeled as 50% monoprotonated and 50% diprotonated, based on the H atom positions, consistent with a formal Co­(III) assignment. The Co–N bond lengths range from 1.918(15) to 1.928(15) Å (Table 1), in agreement with a low-spin Co­(III) center in an approximately octahedral ligand field.^31^ The coordination geometry is close to an ideal octahedral geometry with intraligand bite angles (N–Co–Nintra) of 88.29(6)°, 88.82(6)°, and 90.23(6)°. Suitable single crystals of the Co­(II) analog could not be obtained; therefore, the structure of the related complex, [Co^II^(4-BrPhB­(pz)_3_)_2_] (Figure S5), was used for comparison. In [Co^II^(4-BrPhB­(pz)_3_)_2_], the Co–N bond lengths are significantly longer (2.091(2) to 2.136(2) Å, Table 1) as expected for a Co­(II). The smaller N–Co–N_intra_ angles of 84.22(9)°, 85.04(9)° and 85.25(9)°and nonlinear axial angle (N–Co–N_axial_ = 178.84(11)°) indicate a more distorted coordination environment. For visual comparison, an overlay of [Co^III^(4-PO_3_H_1.5_PhB­(pz)_3_)_2_] and one of the two crystallographically unique [Co^II^(4-BrPhB­(pz)_3_)_2_] molecules is shown in Figure 2 and highlights the substantial structural changes accompanying Co­(II)/Co­(III) interconversion, consistent with large reorganization energy.

**Figure 2:**
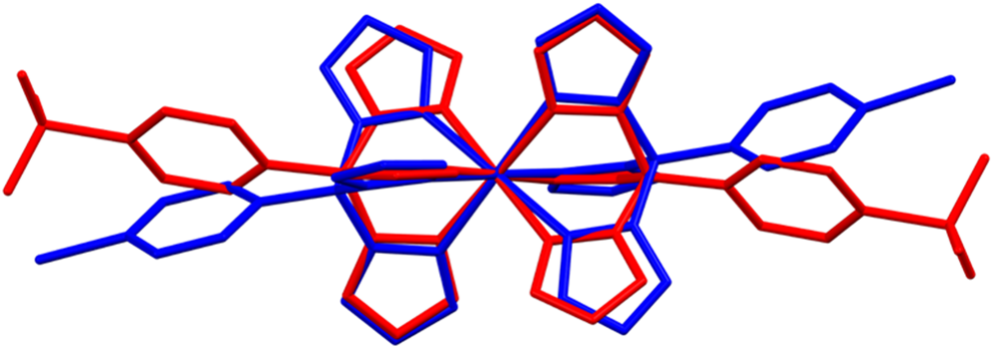
Overlay of crystal structures of [Co^III^(4-PO_3_H_1.5_PhB­(pz)_3_)_2_] (red) with one of the two crystallographically unique [Co^II^(4-BrPhB­(pz)_3_)_2_] molecules (blue). Figure generated using Mercury.^32^

**Table 1:** Selected Bond Distances and Bite Angles for Complexes [Co^III^(4-PO_3_H_1.5_PhB­(pz)_3_)_2_], and [Co^II^(4-BrPhB­(pz)_3_)_2_]

	[Co^III^(4-PO_3_H_1.5_PhB(pz)_3_)_2_]^a^	[Co^II^(4-BrPhB(pz)_3_)_2_]^b^			
		Molecule 1		Molecule 2	
Co–N Distances (Å)	1.9281(15)	2.091(2)	2.136(2)	2.117(2)	2.072(2)
	1.9143(15)	2.131(2)	2.094(2)	2.160(2)	2.114(2)
	1.9181(15)	2.136(2)	2.119(2)	2.081(2)	2.151(2)
Intraligand N–Co–N Angles (°)	88.29(6)	85.04(9)	85.46(9)	85.23(9)	85.02(9)
	90.23(6)	85.25(9)	85.09(9)	85.48(9)	85.82(9)
	88.82(6)	84.22(9)	84.98(9)	85.63(8)	85.66(8)
Trans Angles (°)	180.0	179.51(10)		179.42(10)	
	180.0	179.40(11)		179.21(10)	
	180.0	178.84(11)		179.45(10)	

a Molecule has *C_i_* symmetry so there is only one unique ligand and the trans N–Co–N bond angles are constrained to 180**°**.

b There are two unique molecules in the asymmetric unit, each with C_1_ point symmetry.

Electrochemical analysis of [Co­(4-PO_3_H_2_PhB­(pz)_3_)_2_] was performed by scan-rate-dependent cyclic voltammetry (Figure 3). The Co­(II)/(III) redox couple appears at *E*_1/2_ = −0.64 V vs Fc^+/0^, which is cathodically shifted relative to [Co^II/III^(HB­(pz)_3_)_2_]^0/+^ (−0.50 V vs Fc^+/0^).^20^ The shift is attributed to the presence of the electron-donating 4-PO_3_H_2_Ph substituent on the borate ligand, which increase the electron density at the metal center relative to the parent hydride complex. The peak-to-peak separation (Δ*E*_p_) of 360 mV significantly exceeds that expected for a reversible one-electron redox couple (59–120 mV). The cathodic-to-anodic peak current ratio (*i*_p,c_/*i*_p,a_ = 0.71) further indicates quasi-reversible electron transfer behavior. Such behavior is consistent with other complexes that exhibit CTISC,^20,31^ where large inner-sphere reorganization energy contributes to slow electron transfer kinetics.

**Figure 3:**
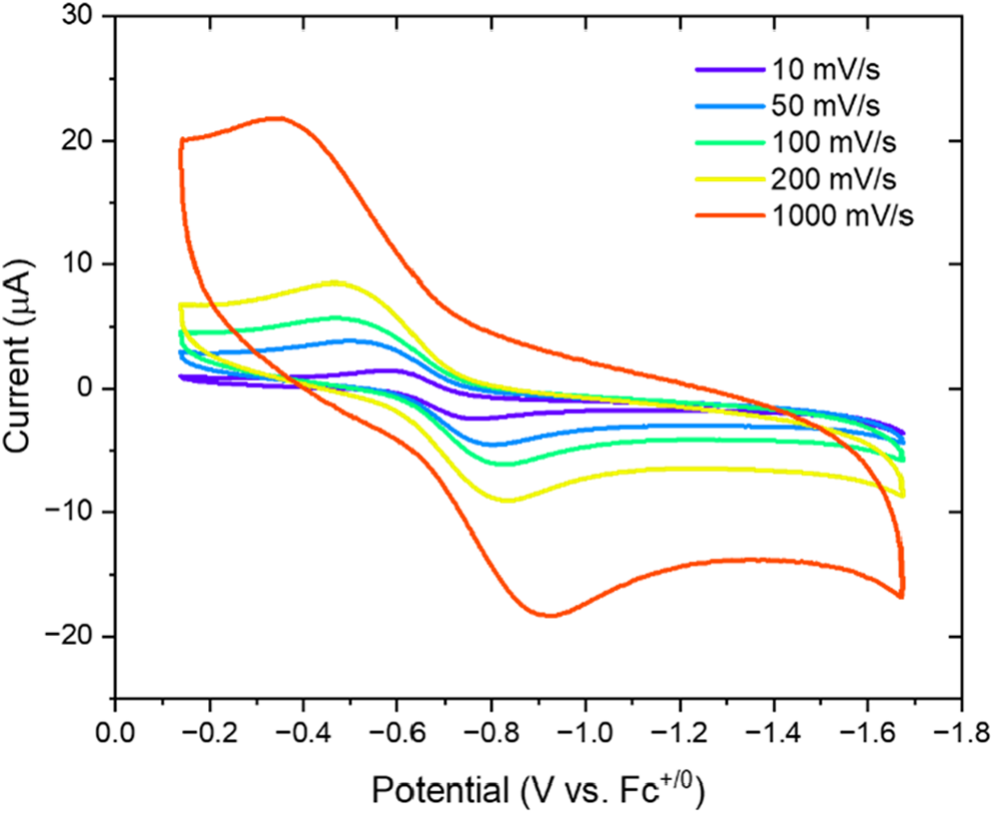
Cyclic voltammetry of [Co­(4-PO_3_H_2_PhB­(pz)_3_)_2_] at scan rates of 10, 50, 100, 200, and 1000 mV/s with 0.1 M TBAPF_6_ as the supporting electrolyte in methanol. *E*_1/2_ = −0.64 V vs Fc^+/0^; Δ*E*_p_= 360 mV; and *i*_p,c_/*i*_p,a_ = 0.71, confirming quasi-reversible behavior.

The magnetic properties of [Co­(4-PO_3_H_2_PhB­(pz)_3_)_2_] were investigated using the Evans method. The measured magnetic susceptibility of Co­(II) is 4.34 μB, consistent with a high-spin configuration (*S* = 3/2), whereas Co­(III) exhibited a magnetic moment of 0.52 μB, indicative of a low-spin state (*S* = 0). These values closely match those reported for the related [Co^II/III^(HB­(pz)_3_)_2_] complex,^20^ confirming [Co­(4-PO_3_H_2_PhB­(pz)_3_)_2_] undergoes CTISC. The similarity between [Co­(4-PO_3_H_2_PhB­(pz)_3_)_2_] and [Co^II/III^(HB­(pz)_3_)_2_] also indicates that the incorporation of the 4-PO_3_H_2_Ph substituent has a limited effect on the spin transition phenomenon.

The photoanode was constructed through a stepwise assembly process involving the sequential absorption of Ru­(II)­P2, Zr^4+^, and [Co­(4-PO_3_H_2_PhB­(pz)_3_)_2_]. At each stage, the successful incorporation of the respective components was confirmed using complementary spectroscopic techniques. Attachment of Ru­(II)­P2 binding was confirmed with electronic absorption spectroscopy, where the characteristic MLCT associated with the Ru polypyridyl compound was observed (Figure 4). Subsequent binding of Zr^4+^ did not alter the absorption spectrum. Therefore, X-ray photoelectron spectroscopy (XPS) was used to confirm the presence of Zr^4+^. As shown in Figure S6, a 0.13 eV shift in the phosphorus 2p peak provides clear evidence for Zr^4+^ coordination. Introduction of [Co­(4-PO_3_H_2_PhB­(pz)_3_)_2_] resulted in distinct changes in the absorption spectrum consistent with incorporation, as determined by comparison to the [Co­(4-PO_3_H_2_PhB­(pz)_3_)_2_] solution spectrum, Figure 4. An elemental XPS survey confirmed the presence of Ru^2+^, Zr^4+^ and Co^2+^ on the TiO_2_. Quantitative analysis by ICP-MS revealed a Ru:Zr:Co ratio of 2.5:1:0.2 (Table S2), indicating incomplete but sequential functionalization. The reduced loading of [Co­(4-PO_3_H_2_PhB­(pz)_3_)_2_] is attributed to steric hindrance imposed by Ru­(II)­P2, which likely limited the subsequent binding of Zr^4+^ and [Co­(4-PO_3_H_2_PhB­(pz)_3_)_2_].^30^ Despite the lower surface coverage, the presence of [Co­(4-PO_3_H_2_PhB­(pz)_3_)_2_] was sufficient to observe regeneration behavior in transient spectroscopic measurements, as discussed below.

**Figure 4:**
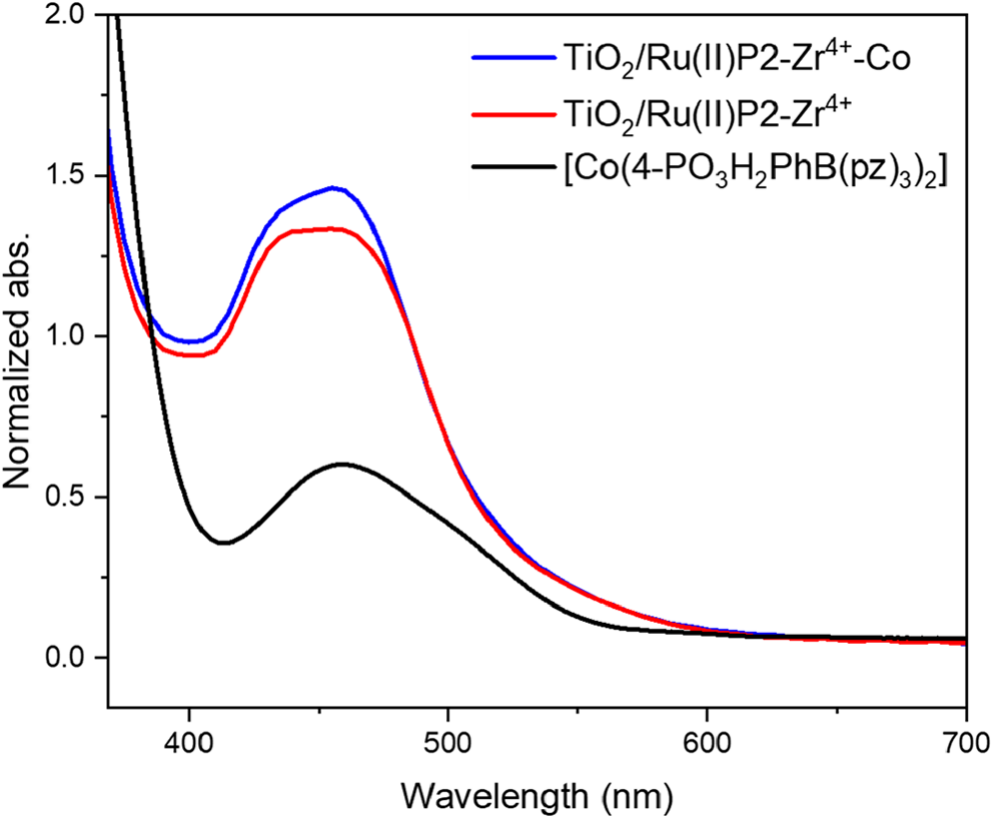
UV–vis of TiO_2_/Ru­(II)­P2–Zr^4+^-Co, TiO_2_/Ru­(II)­P2–Zr^4+^, and [Co­(4-PO_3_H_2_PhB­(pz)_3_)_2_] in methanol.

With the photoanode constructed, the focus shifted to characterizing the relevant charge transfer processes with time-resolved spectroscopy. The key photophysical and photochemical processes are outlined in Scheme 1. The arrows describe the electron transfer process, with the numbers indicating the chronological order of the processes. Upon photoexcitation, the Ru­(II)­P2 sensitizer is excited to a single metal-to-ligand charge-transfer excited state (^1^MLCT*, arrow 1). Ultrafast intersystem crossing populates the triplet excited state (^3^MLCT*, arrow 2), which is capable of injecting an electron into the conduction band of TiO_2_ (arrow 3). After electron injection, the oxidized chromophore, Ru­(III), is generated. Ru­(III) can undergo one of two competing processes (1) regeneration by [Co­(4-PO_3_H_2_PhB­(pz)_3_)_2_] (arrow 4) or (2) back electron transfer with electrons in the TiO_2_ conduction band (arrow 5). If regeneration is rapid, the ultimate recombination pathways would also include recombination to the oxidized cobalt center (Co­(III)) (arrow 6). Because the surface coverage of [Co­(4-PO_3_H_2_PhB­(pz)_3_)_2_] is incomplete, both recombination pathways are operative (arrow 5 and arrow 6).

**Scheme 1:**
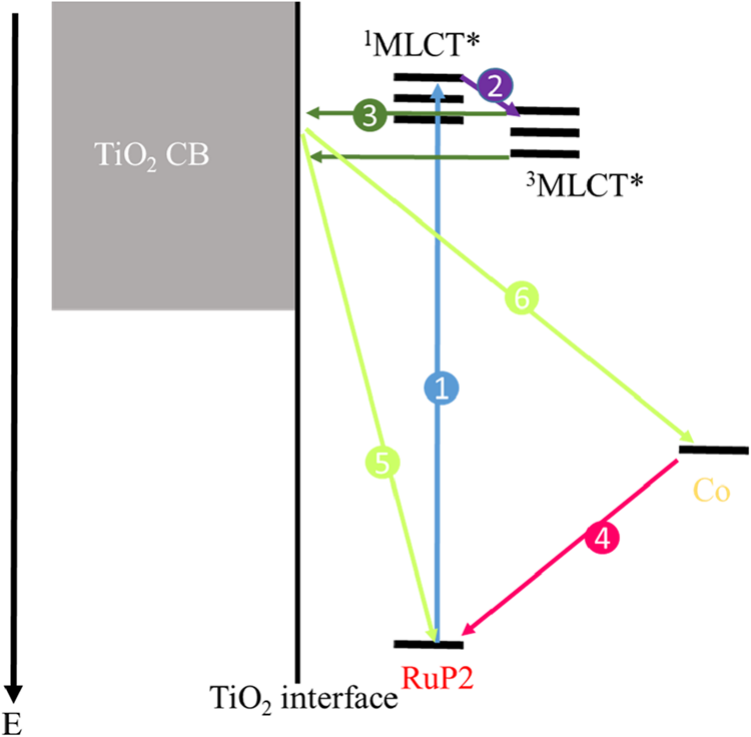
Schematic Diagram Illustrating the Electron Transfer Processes in the TiO_2_/Ru­(II)­P2–Zr^4+^-Co Construct

Ultrasfast transient absorption spectroscopy was used to probe the early photoinduced process (Scheme 1, arrow 1–4). Immediately following photoexcitation, a ground-state bleaching between 420 and 560 nm (λ_max_ = 455 nm) and two excited state absorption features: one between 350 and 400 nm (λ_max_ = 380 nm) and another extending from 560 nm to the near IR were observed (Figure 5A). These features are consistent with the formation of the Ru­(II)­P2 excited state.^33^ The temporal evolution of the excited state absorption at 380 nm is consistent with injection from the ^3^MLCT* into the TiO_2_ conduction band (Figure 5B). Kinetic analysis at 380 nm revealed two time constants, 29 ± 5 and 361 ± 34 ps, which closely match those measured for the Ru­(II)­P2/TiO_2_ control (25 ± 2 and 316 ± 22 ps). In contrast, the excited-state lifetime of Ru­(II)­P2 in homogeneous solution is much longer (423 ± 2.9 ns), confirming electron injection into TiO_2_ is dominant. The similarity in excited-state lifetimes between the complete photoanode and the Ru­(II)­P2/TiO_2_ control further supports that energy transfer is negligible.

**Figure 5:**
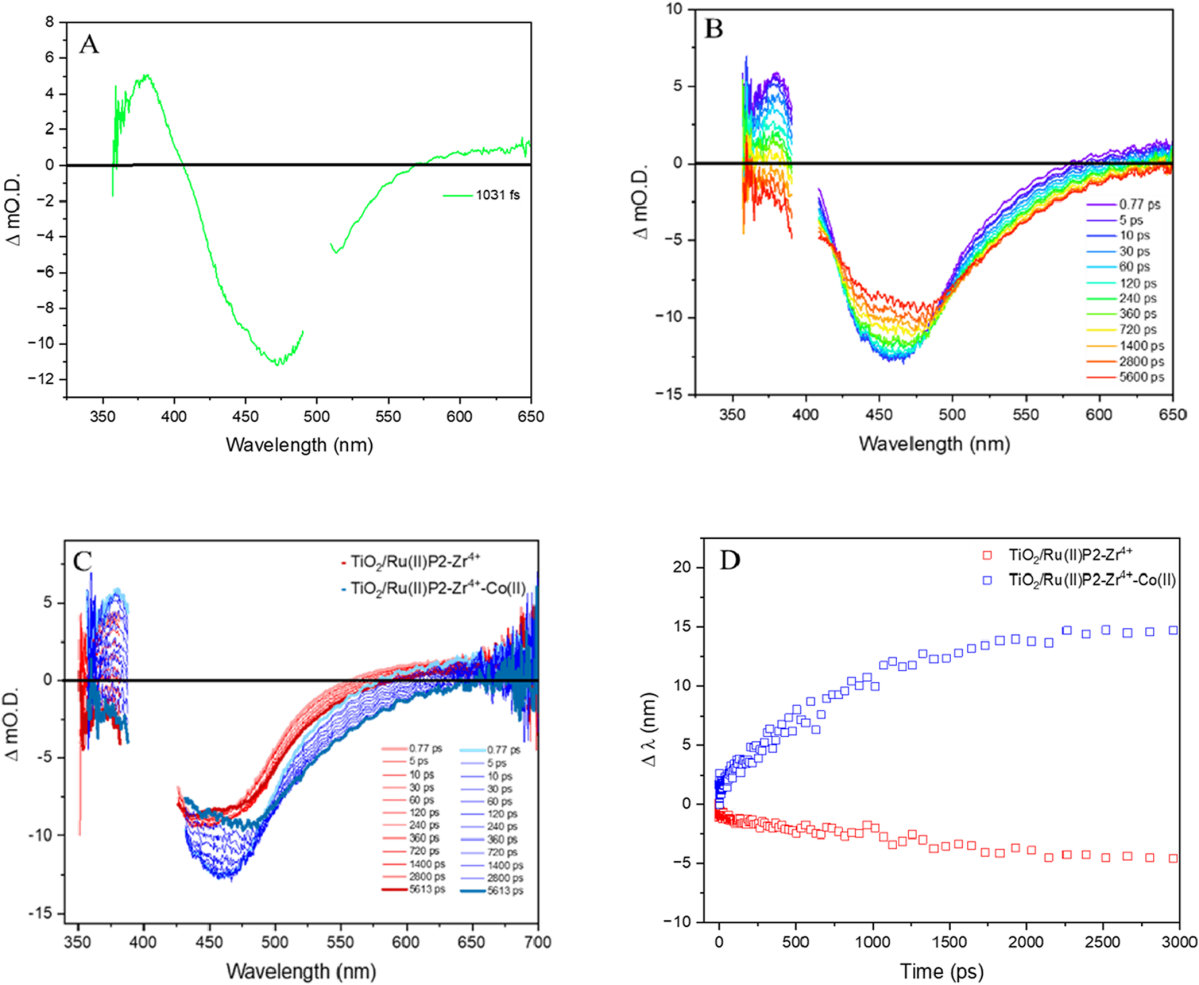
(A) Ultrafast transient absorption spectrum of TiO_2_/Ru­(II)­P2–Zr^4+^-Co­(II) from 0.5 to 2.17 ps, Excitation wavelength: 500 nm; The pump region is removed for clarity. (B) Ultrafast transient absorption spectrum of TiO_2_/Ru­(II)­P2–Zr^4+^-Co­(II) from 0.77 ps to 5.6 ns. Excitation wavelength: 400 nm; (C) overlap of ultrafast transient absorption spectrum TiO_2_/Ru­(II)­P2–Zr^4+^ and TiO_2_/Ru­(II)­P2–Zr^4+^-Co­(II), from 0.77 ps to 5.6 ns. Excitation wavelength: 400 nm; (D) wavelength shifting of the ground state bleach’s peak as a function of time for TiO_2_/Ru­(II)­P2–Zr^4+^ and TiO_2_/Ru­(II)­P2–Zr^4+^-Co­(II).

At longer delay times, distinct differences emerge between the complete photoanode and sensitizer/TiO_2_ control, indicative of the regeneration of Ru­(III)­P2 by Co­(II) (Figure 5C). Specifically, a red shift in the ground state bleach is observed, from 455 nm (λ_max_ of Ru­(II)­P2) to 480 nm (λ_max_ of [Co­(4-PO_3_H_2_PhB­(pz)_3_)_2_]). To visualize the kinetics of the electron regeneration process, the shift in absorption was plotted as a function of time (Figure 5D). The lifetime of the shift was found to be 802 ± 16 ps (*k*_et_ = 1.24 × 10^9^ ± 2.76 × 10^7^ s^–1^) (Figure S7). For comparison, a previously reported Ru_a_-Zr^4+^-Ru_b_ photoanode exhibited a faster regeneration rate of 5.90 × 10^9^ s^–1^.^34^ The slower kinetics observed here are consistent with the large inner-sphere reorganization energy associated with the CTISC process, which imposes an additional kinetic barrier. A concept that supports the use of highly reversible (in terms of electrochemical behavior) mediators in DSSC, a strategy exemplified by Hamann.^35^

To probe the recombination kinetics, nanosecond transient absorption was employed to monitor electron transfer from the TiO_2_ to the oxidized species, Ru­(III)­P2 and Co­(III) (Figure 6A,B). The initial ground-state bleach observed in the transient spectra confirms the presence of a mixture of Co­(III) and Ru­(III), consistent with the ultrafast measurements and confirming the presence of two recombination centers. Recovery of the bleach reflects the recombination of conduction-band electrons with the oxidized chromophore and mediator, and the restoration of the photoanode’s initial state. Recombination kinetics in sensitized metal oxide assemblies are commonly described using a stretched exponential model to account for the distribution of trap states within the semiconductor (eqs 1–3).^36^ Accordingly, the data of the Ru­(II)­P2/TiO_2_ control was fitted to a stretched exponential with a characteristic lifetime (τ_rec_) of 42 ± 2.1 μs (Figure 6C and S8). In contrast, the full TiO_2_/Ru­(II)­P2–Zr^4+^-Co­(II) assembly exhibited slower recombination kinetics, with τ_rec_ = 52 ± 1.0 μs (Figure 6C and S8). The increase in lifetime indicates that the incorporation of [Co­(4-PO_3_H_2_PhB­(pz)_3_)_2_] effectively suppresses charge recombination. Notably, despite the lower surface coverage of [Co­(4-PO_3_H_2_PhB­(pz)_3_)_2_] relative to Ru­(II)­P2 (approximately 1:10), the impact of the recombination kinetics was significant (30% increase), highlight the influence of the CTISC process.

1 $$\Delta \mathrm{OD}\left(t\right)={\Delta \mathrm{OD}}_{t=0}e^{-{\left(t/\tau_{\mathrm{ww}}\right)}^{\beta }}$$

2 $$\tau_{avg}=\frac{\tau_{ww}}{\beta }\Gamma \left(\frac{1}{\beta }\right)$$

3 $$\Gamma =\int_{0}^{\infty }u^{x-1}e^{-u}du$$

**Figure 6:**
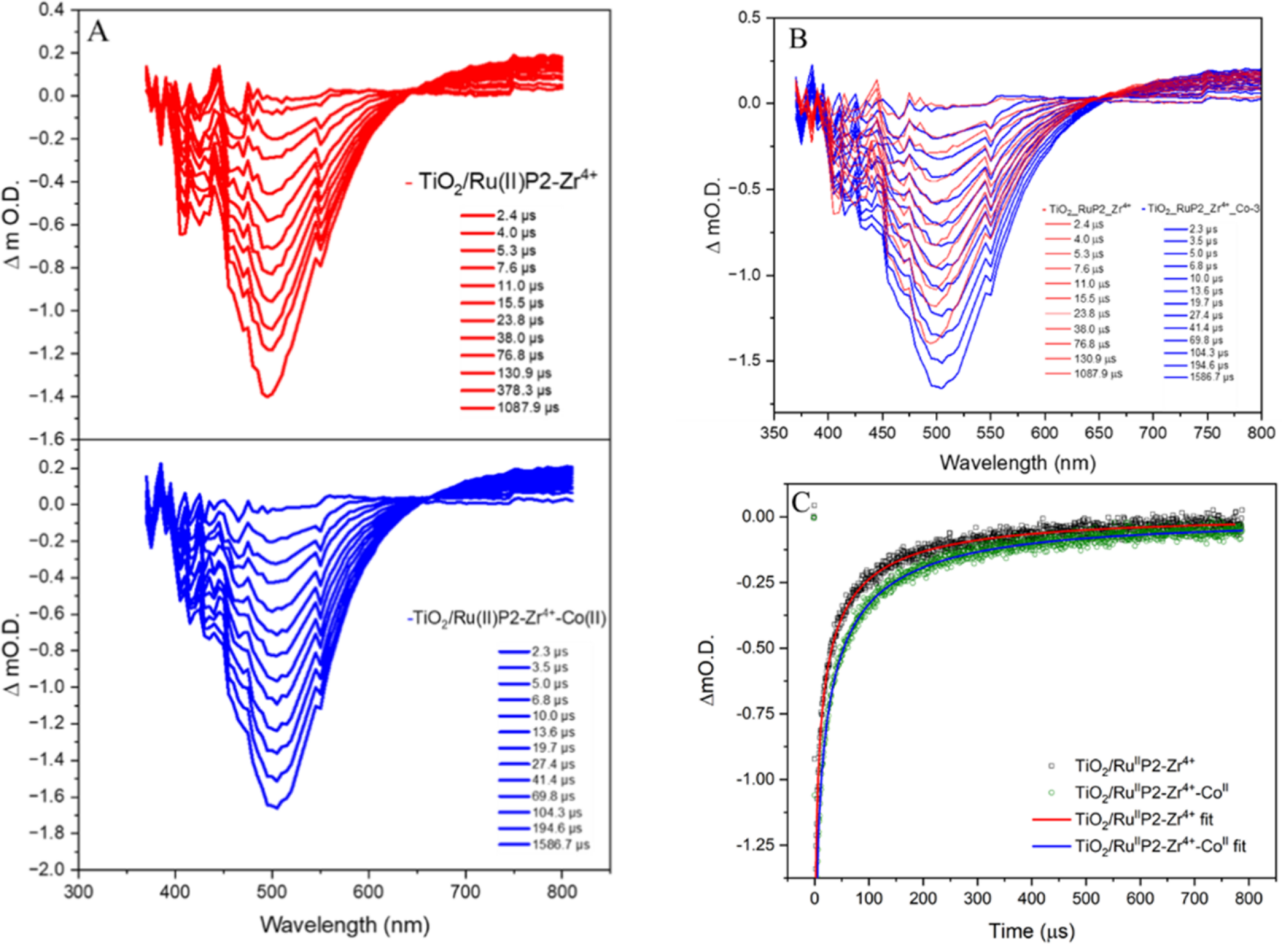
(A) Transient absorption spectra of TiO_2_/Ru­(II)­P2–Zr^4+^ and TiO_2_/Ru­(II)­P2–Zr^4+^-Co­(II), with 532 nm excitation. (B) Overlap of spectra for TiO_2_/Ru­(II)­P2–Zr^4+^ and TiO_2_/Ru­(II)­P2–Zr^4+^-Co­(II) with 532 nm excitation. (C) Single wavelength kinetic decay of TiO_2_/Ru­(II)­P2–Zr^4+^ and TiO_2_/Ru­(II)­P2–Zr^4+^-Co­(II). The kinetic decay were binned for better clarity. The raw kinetic decay traces are shown in Figure S8.

The spin state preferences of cobalt center were evaluated by DFT calculations that compared the relative energies of the high-spin and low-spin configurations. For Co­(II), the low-spin state is calculated to be higher in energy than that of the high-spin state by approximately 0.2 eV, corresponding to an equilibrium constant of ∼3.6 × 10^–4^. The small equilibrium constant indicates a strong thermodynamic preference for the high-spin configuration, consistent with the experimentally measured magnetic susceptibility. In contrast, Co^III^ exhibits a substantially larger energetic difference between the spin states, with the low-spin configuration favored by approximately 1.64 eV. The pronounced stabilization of low-spin Co­(III) is well documented experimentally and aligns with the stronger ligand field experienced by the higher oxidation state cobalt ion.^37,38^ Combining the reorganization energies associated with oxidation and spin-state transitions provides insight into the overall reorganization energy involved in the CTISC process. The total reorganization energy, calculated in accordance with established methodology,^14^ is 3.74 eV. Of this value, approximately 2.40 eV originates from the inner-sphere reorganization.

The lifetimes of the charge-separated states were further evaluated through open-circuit voltage decay measurements performed on the assembled photoelectrodes. Under the steady-state illumination, no net current flows to the external circuit, and the open circuit voltage, *V*_oc_, is set by the population of excess electrons in the TiO_2_ conduction band. After the removal of the light source, charge recombination dominates, and the decay in *V*_oc_ reflects the kinetics of electron recombination with oxidized species at the interface. The electron lifetime (τ_e_) can be extracted from the *V*_oc_ decay according to the formalism derived by Zaban et al., where τ_e_ is inversely proportional to the slope of the *V*_oc_ vs time relationship (eq 4,^39^ where *k*_b_ is the Boltzmann constant; *T* is the temperature; *V*_oc_ is the open circuit voltage; *e* is the electron charge, and *t* is time). Representative open circuit voltage decay traces are shown in Figure 7. Comparison of the decay kinetics revealed that τ_e_ increases from TiO_2_/Ru­(II)­P2–Zr^4+^ (slope = −13.86 ± 1.60) to TiO_2_/Ru­(II)­P2–Zr^4+^-Co­(II) (slope = −8.03 ± 0.67), indicating a longer-lived charge-separated state in the presence of the cobalt complex. The results are consistent with the transient absorption data and support the conclusion that incorporation of the CTISC mediator effectively suppresses recombination.

4 $$\tau_{e}=-\frac{k_{b}T}{e}{\left(\frac{dV_{\mathrm{oc}}}{dt}\right)}^{-1}$$

**Figure 7:**
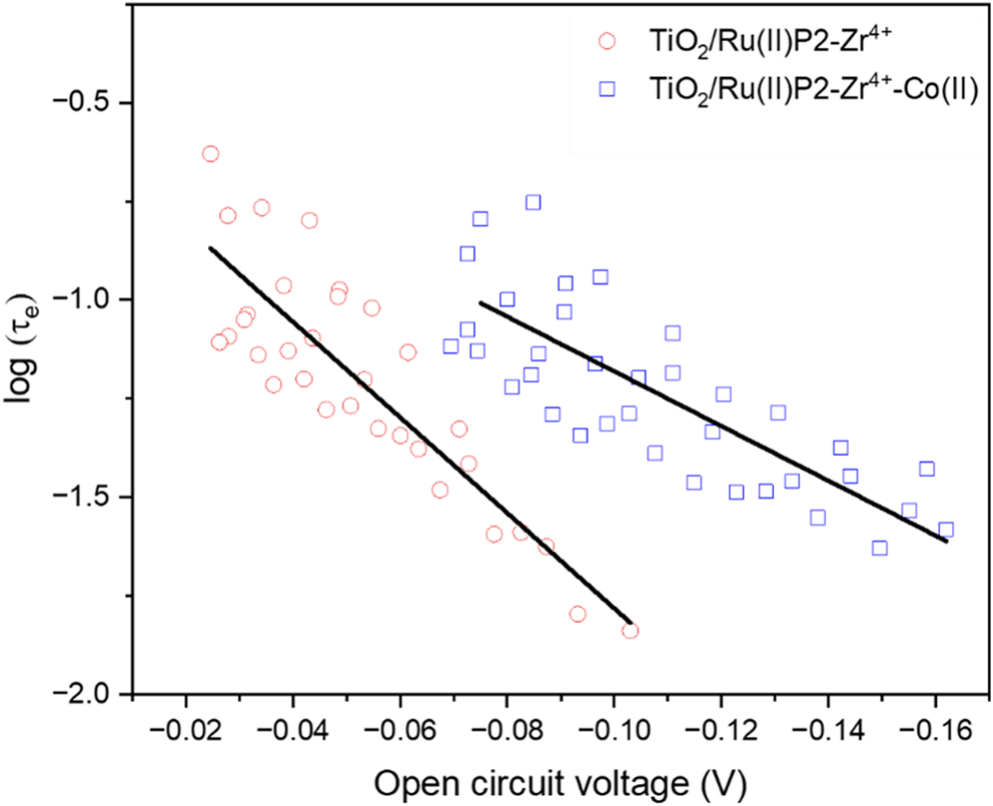
Open-circuit voltage decay measurements of TiO_2_/Ru­(II)­P2–Zr^4+^ and TiO_2_/Ru­(II)­P2–Zr^4+^-Co­(II) after 0.2 Sun illumination. Linear fits of the data are overlaid.

## Conclusion

In this study, we demonstrated that the inner-sphere reorganization energy of a cobalt-based CTISC complex provides an effective strategy for controlling the lifetime of charge-separated states in self-assembled photoanodes. Transient absorption spectroscopy revealed that regeneration and recombination kinetics are strongly influenced by the large structure reorganization associated with the Co-centered redox process. The regeneration rate of the Ru-based sensitizer by the cobalt complex is slightly slower than that observed for other related studies of ruthenium-based catalyst assemblies due to the inner-sphere reorganization accompanying the Co­(II/III) redox transition. Importantly, incorporation of the CTISC complex into the photoanode leads to a measurable enhancement in charge-separated state lifetime, with an approximately 30% increase relative to control anodes lacking the cobalt donor. Density functional theory calculations further support this behavior, showing that the large reorganization energy arises from the pronounced structural and electronic difference between the high-spin Co­(II) and low-spin Co­(III) states. Together, these results demonstrate that controlling spin-state-dependent reorganization provides a powerful strategy for modulating charge recombination kinetics and advances the design of efficient, self-assembled photoelectrochemical architectures.

## Supplementary Material



## Notes

Synthesis schemes of [Ru­(bpy)­(4,4′-(PO_3_H_2_)_2_bpy)_2_]­Cl_2_ and [Co­(4-PO_3_H_2_PhB­(pz)_3_)_2_], ^1^H NMR of [Ru­(bpy)­(4,4′-(PO_3_H_2_)_2_bpy)_2_]­Cl_2_ and [Tl­(4-BrPhB­(pz)_3_)], mass spectrometry of [Co­(4-PO_3_H_2_PhB­(pz)_3_)_2_], crystal structure of [Co­(III)­(4-PO_3_H_1.5_PhB­(pz)_3_)_2_]·2H_2_O and [Co­(II)­(4-BrPhB­(pz)_3_)_2_]·2DCM, crystal data and structure refinement for [Co­(III)­(4-PO_3_H_1.5_PhB­(pz)_3_)_2_] and [Co­(II)­(4-BrPhB­(pz)_3_)_2_], XPS of high resolution scan of phosphorus of TiO_2_/Ru­(II)­P2 and TiO_2_/Ru­(II)­P2–Zr^4+^, elemental analysis of desorbed photoanode constructs via ICP-MS (Ru, Zr and Co), Exponential fitting of wavelength shifting of GSB’s peak as function of time of TiO_2_/Ru­(II)­P2–Zr^4+^-Co­(II) (PDF)
