# Supplementary material for: Long-Lived Charge-Separated States in Self-Assembled TiO2 Photoanodes Incorporating a Spin-Transition Cobalt Complex
Source: J Phys Chem C Nanomater Interfaces. 2026 Mar 26;130(14):5192–201. doi: 10.1021/acs.jpcc.6c00432 (PMC13071918; doi:10.1021/acs.jpcc.6c00432)
Supplement: Supplementary file 1 [file jp6c00432_si_001.pdf]

Supporting Information

Long-Lived Charge-Separated States in Self-Assembled TiO_2 Photoanodes Incorporating a Spin-Transition Cobalt Complex

Tzu-Ching Cheng†,Vasily Vorobyev‡, Savannah Pearson‡, Niroshani S. Abeynayake‡,

Carla Slebodnick‡, Zhichun Shi‡, and Amanda J. Morris†‡*

†Department of Materials Science & Engineering, Virginia Tech, Blacksburg, Virginia 24061, United States

‡Department of Chemistry, Virginia Tech, Blacksburg, Virginia 24061, United States

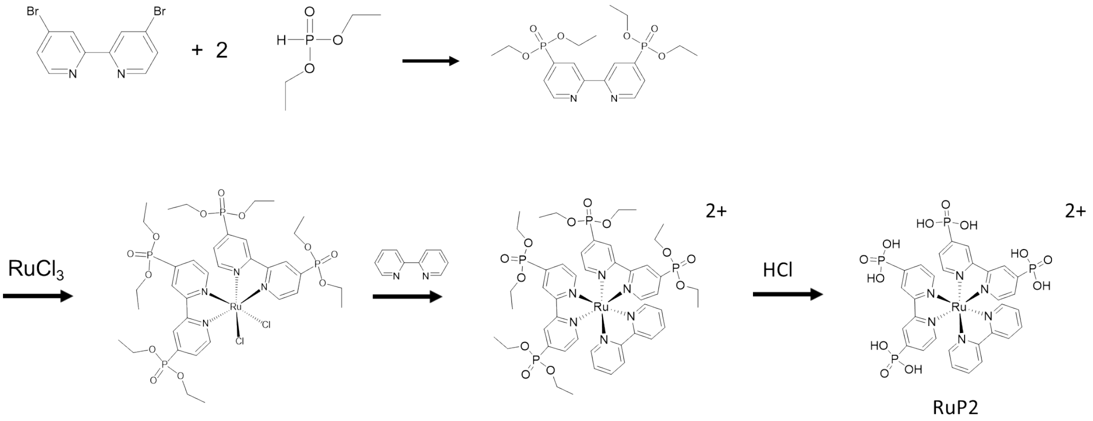

Scheme S1. Synthesis scheme of [Ru(bpy)(4,4'-$(PO_3H_2)_2$bpy)$_2$](Cl)$_2$

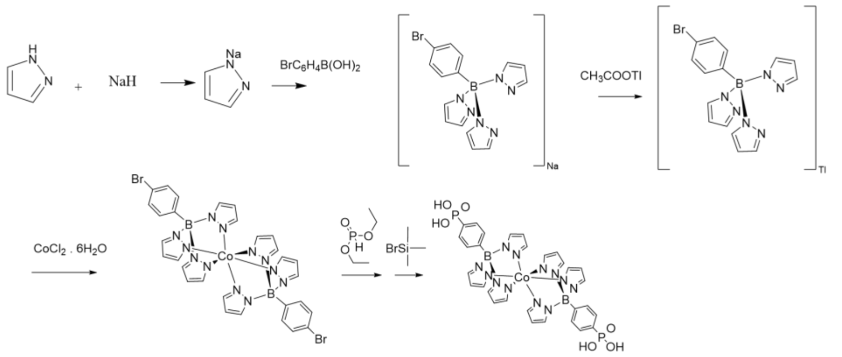

Scheme S2. Synthesis scheme of [Co(4-PO_3H_2PhB(pz)$_3$)$_2$]

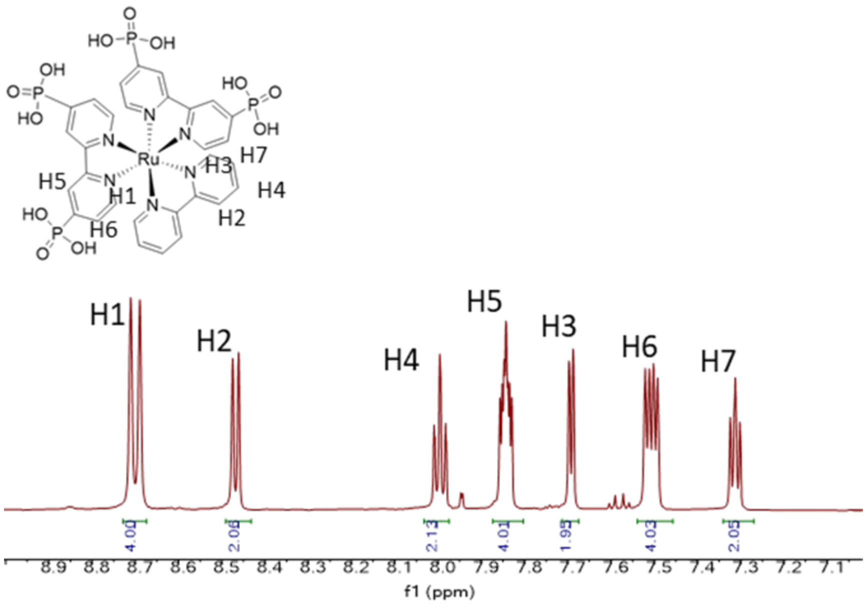

Figure S1. ^{1}**H NMR of synthesizing [Ru(bpy)(4,4'-(PO$_3$H$_2$)$_2$bpy)$_2$](Cl)$_2$**

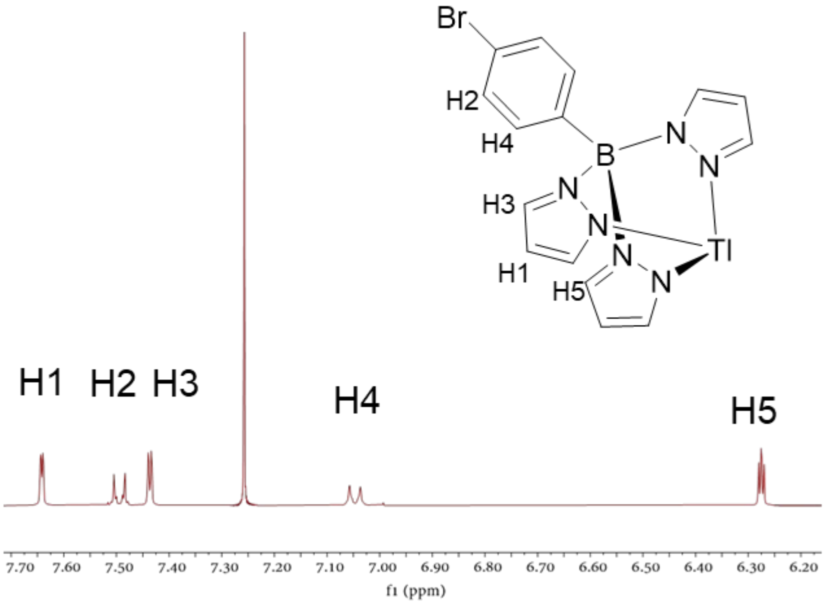

Figure S2. ^{1}**H NMR of Thallium (4-bromophenyl)tris(l-pyrazolyl)borate in CDCl$_3$**

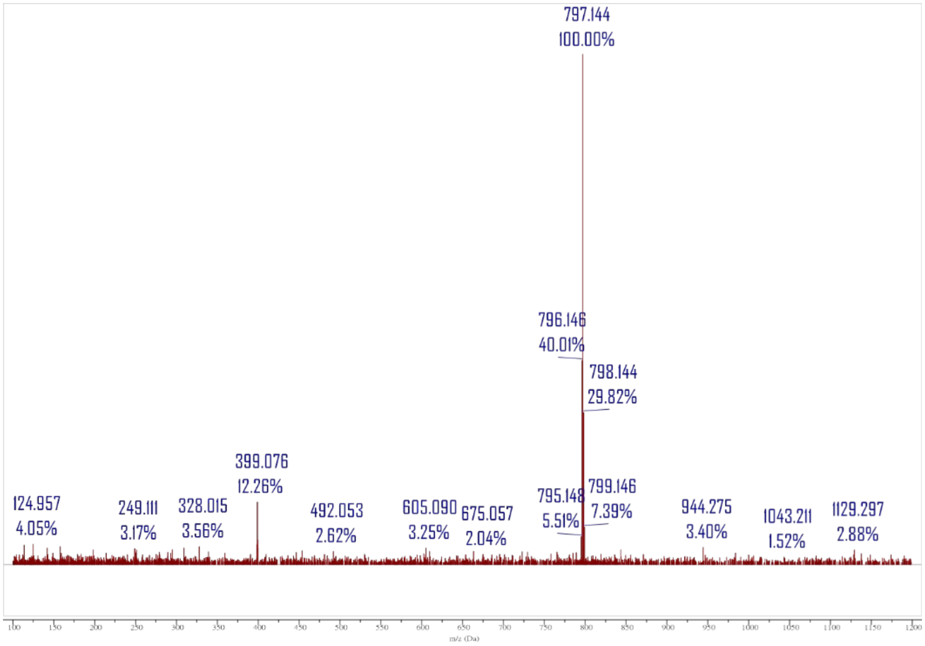

Figure S3. Mass spectrometry of [Co(4-PO_3H_2PhB(pz)$_3$)$_2$] (m/z calcd: 797.1487, found: 797.1401 [M]$^+$)

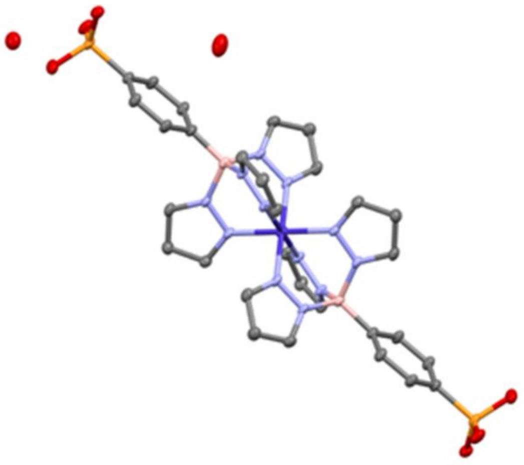

Figure S4. Crystal structure of [Co^{III}(4-$PO_3H_{1.5}$PhB(pz)$_3$)$_2$]·2H_2O with anisotropic displacement ellipsoids draw at 50% probability. Violet: Co, lavender: N , pale pink: B, gray: C, orange: P and red: O. H was omitted for clarity.

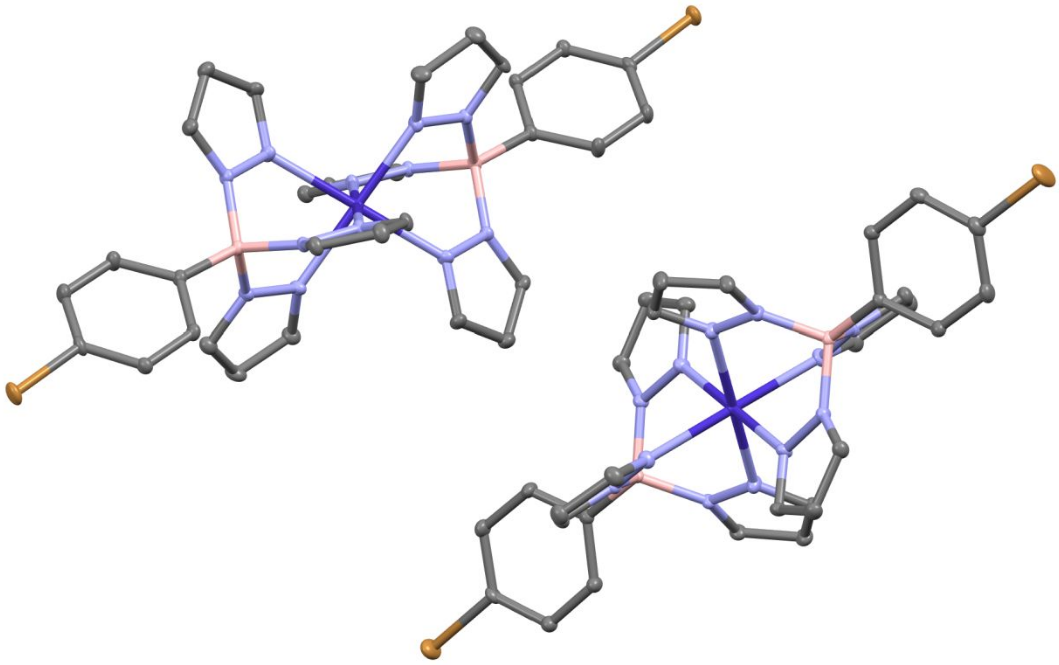

Figure S5. Crystal structure of $[Co^{II}(4\text{-}BrPhB(pz)_3)_2]\cdot 1.5DCM$ with anisotropic displacement ellipsoids draw at 50% probability. Violet: Co, lavender: N , pale pink: B, gray: C, and canary yellow: Br. The DCM solvate and H-atoms were omitted for clarity.

Table S1. Crystal data and structure refinement for $[Co^{III}(4\text{-}PO_3H_{1.5}PhB(pz)_3)_2]$ and $[Co^{II}(4\text{-}BrPhB(pz)_3)_2]$

compound	$[Co^{III}(4\text{-}PO_3H_{1.5}PhB(pz)_3)_2]$	$[Co^{II}(4\text{-}BrPhB(pz)_3)_2]$.1.5DCM
Empirical formula	$C_{30}H_{37}B_2CoN_{12}O_{10}P_2$	$C_{30}H_{26}B_2Br_2CoN_{12}$ .1.5(CH_2Cl_2)
Formula weight	868.20	922.39
Temperature/K	110.00(10)	100.00(11)
Crystal system	triclinic	triclinic
Space group	P-1	P1
a/Å	8.52570(10)	11.59880(10)
b/Å	8.59600(10)	11.94050(10)
c/Å	13.0277(2)	13.41860(10)
α/°	108.2590(10)	80.5620(10)
β/°	91.1470(10)	88.9720(10)
γ/°	93.8980(10)	89.5480(10)
Volume/Å^3	903.73(2)	1832.94(3)
Z, Z’	1, 0.5	2, 2

ρ_{calc}g/cm^3	1.595	1.671
μ/mm^{-1}	5.210	2.910
F(000)	448.0	920.0
Crystal size/mm^3	0.12 × 0.1 × 0.02	0.34 × 0.175 × 0.138
Radiation	Cu Kα (λ = 1.54184)	Mo Kα (λ = 0.71073)
2Θ range for data collection/°	7.152 to 148.888	6.086 to 76.414
Index ranges	-10 ≤ h ≤ 9, -10 ≤ k ≤ 10, -16 ≤ l ≤ 15	-20 ≤ h ≤ 19, -20 ≤ k ≤ 20, -23 ≤ l ≤ 23
Reflections collected	22782	91230
Independent reflections	3675 [R_{int} = 0.0338, R_{sigma} = 0.0216]	37511 [R_{int} = 0.0326, R_{sigma} = 0.0453]
Data/restraints/parameters	3675/0/283	37511/3/929
Goodness-of-fit on F^2	1.065	1.031
Final R indexes [I>=2σ (I)]	R_1 = 0.0333, wR_2 = 0.0873	R_1 = 0.0343, wR_2 = 0.0826
Final R indexes [all data]	R_1 = 0.0340, wR_2 = 0.0878	R_1 = 0.0443, wR_2 = 0.0854
Largest diff. peak/hole / e Å^{-3}	0.36/-0.33	1.45/-0.85

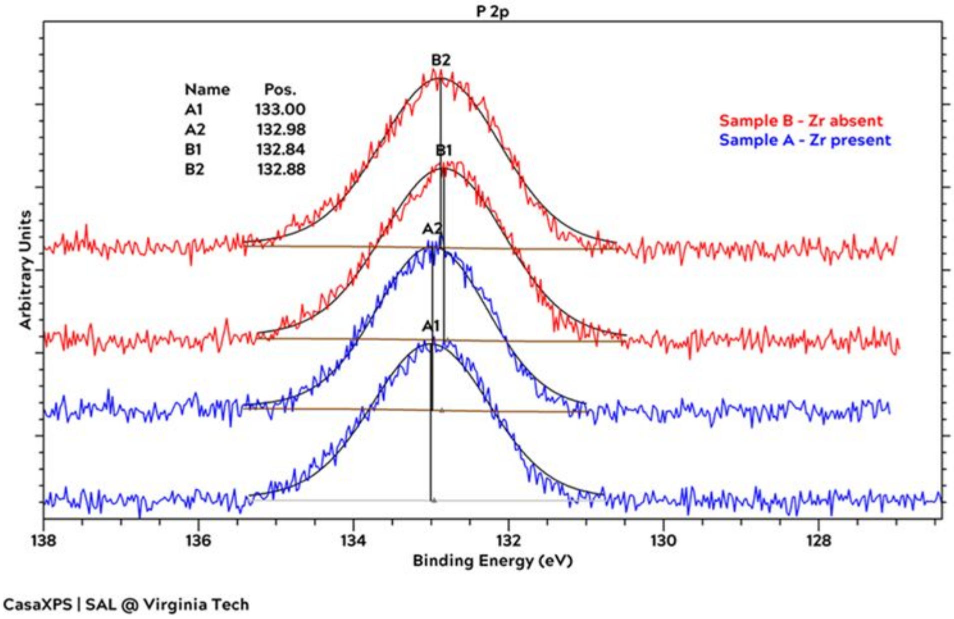

Figure S6. High resolution scan of phosphorus with Zr $^{4+}$(blue)and without Zr $^{4+}$ (red) bonding

Table S2. ICP-MS of desorbed photoanode constructs

Atom concentration (in ppm)	Ru	Zr	Co
TiO_2/RuP2-Zr^{4+}-Co	25.8	10.7	2.3

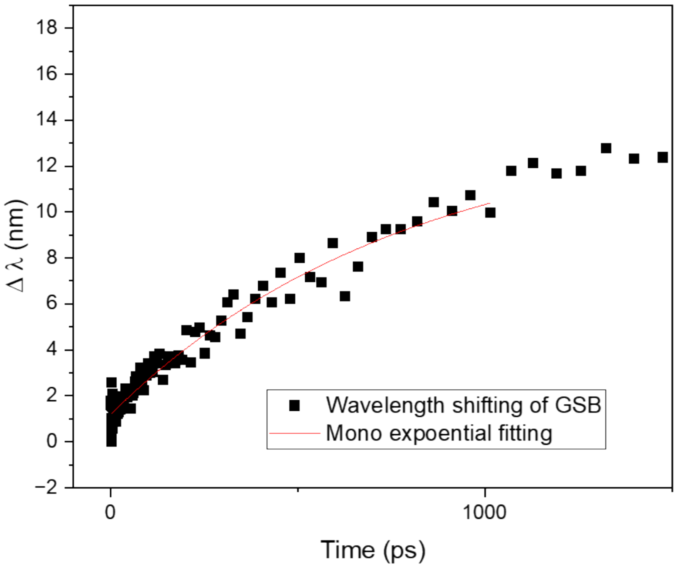

Figure S7. Wavelength shifting of GSB's peak as a function of time of TiO_2/RuP2-Zr^{4+}-Co with single exponential fitting trace (red) within first 1000 ps with τ= 802 ± 16 ps and R^2 of 0.98.

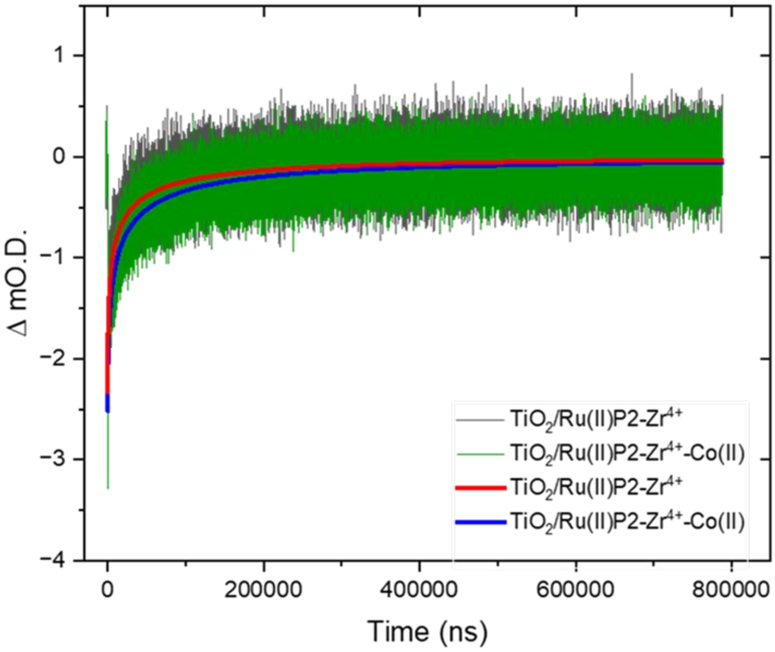

Figure S8. Single wavelength kinetic decay of TiO_2/Ru(II)P2-Zr^{4+} and TiO_2/Ru(II)P2-Zr^{4+}-Co(II) were fitted to stretched exponential function with TiO_2/Ru(II)P2-Zr^{4+} : τ_{avg}= 42 ±2.1 µs, stretching exponent (β) value of 0.303±0.023 and R^2 of 0.44; TiO_2/Ru(II)P2-Zr^{4+} -Co(II) : τ_{avg} = 52±1.0 µs, β of 0.287±0.017 and R^2 of 0.60.

To obtain the best fit for the TiO_2/Ru(II)P2-Zr^{4+}-Co(II) kinetic trace, a bi-stretched exponential function was employed. However, the fitting results were nearly identical to those obtained using a single stretched exponential function. Therefore, the confidence level in the obtained parameters was weak. We speculate that the intrinsic nature of the stretched exponential function, particularly the long tail toward the end of the decay, effectively captures this minor component.
